# The Utility of Responsive Neurostimulation for the Treatment of Pediatric Drug-Resistant Epilepsy

**DOI:** 10.3390/brainsci13101455

**Published:** 2023-10-13

**Authors:** Martin G. Piazza, Gregory Varga, William Welch, Taylor J. Abel

**Affiliations:** 1 Department of Neurological Surgery, University of Pittsburgh, Pittsburgh, PA 15260, USA; piazzamg@upmc.edu (M.G.P.); varga.gregory@medstudent.pitt.edu (G.V.); 2 Department of Neurology, University of Pittsburgh, Pittsburgh, PA 15260, USA; welchwp@upmc.edu

**Keywords:** drug-resistant epilepsy, responsive neurostimulation, neuromodulation, closed-loop technology, seizure, epilepsy surgery, deep brain stimulation, seizure network

## Abstract

Drug-resistant epilepsy (DRE) has a strongly negative impact on quality of life, as well as the development of pediatric patients. Surgical treatments have evolved over time, including more invasive craniotomies for resection or disconnection. More recently, neuromodulation techniques have been employed as a less invasive option for patients. Responsive neurostimulation (RNS) is the first closed-loop technology that allows for both treatment and device data collection, which allows for an internal assessment of the efficacy of treatment. This novel technology has been approved in adults and has been used off label in pediatrics. This review seeks to describe this technology, its history, and future directions.

## 1. Introduction

Epilepsy is one of the most common neurologic conditions effecting an estimated 1% of children worldwide [1,2,3]. Broadly, epilepsy refers to a clinical condition in which a patient has recurrent, unprovoked seizures, resulting from abnormal brain connectivity networks. Medical management fails to achieve seizure freedom in up 30–40% of these individuals [1,2,4,5,6]. Patients who fail “adequate trials of two tolerated and appropriately chosen and used [anti-epileptic drug] schedules (whether as monotherapies or in combination) to achieve sustained seizure freedom” [4] are considered to have drug-resistant epilepsy (DRE) [4,6,7]. Additional medications have a less than 5% chance of improving seizure control [4,8]. Patients with DRE are at higher risk for significant morbidities, adverse drug reactions, developmental delay, and even death [9,10]. Thus, early referral to surgery for DRE is recommended by the International League Against Epilepsy (ILAE). Early surgical referral is supported by multiple clinical studies [11,12,13] to minimize the numerous quality-of-life and cost implications for these children [3].

As such, epilepsy surgery is an important treatment strategy for patients with DRE. Surgical resection or disconnection of the epileptogenic zone (EZ) from normal brain may be curative for some patients. However, when the EZ overlaps with eloquent areas of cortex, such as the motor or visual areas, resection can lead to unacceptable functional deficits [7]. Advancements in neuromodulation techniques provide alternative approaches without the morbidity of tissue resection. With these treatments, desynchronization of seizure networks can occur via the stimulation of the seizure onset zone (SOZ) itself or indirectly by the stimulation of propagation points within the network, such as the thalamus [14]. 

Responsive neurostimulation (RNS) is the first closed-loop technology offered that both monitors electrical activity and delivers targeted stimulation to the SOZ in response to detected electrographic patterns believed to represent seizure [15]. As DRE is a severe disease with numerous quality-of-life and cost implications for children, increasing available treatment options is imperative [3]. This review focuses on the clinical indications, outcomes, and other technical considerations of RNS as it applies to the pediatric population. 

## 2. Common Neuromodulation Techniques

Neuromodulation strategies vary based on the patient seizure type and frequency, including those that target the SOZ and those that target neural networks associated with seizure propagation [16,17,18]. There are currently three types of devices: vagus nerve stimulation (VNS), deep brain stimulation (DBS), and RNS [19,20]. 

VNS functions by stimulating the vagus nerve in the neck. The proposed mechanism of this device is to modulate epileptogenesis by indirectly increasing the production of norepinephrine and serotonin, which are believed to have anti-seizure effects [16,21]. VNS is reported to reduce generalized seizure frequency by 50% in approximately 60% of patients [22]. DBS consists of bilateral electrodes typically placed in the anterior or centromedian nuclei of the thalamus [23]. Stimulation to these regions of the thalamus is thought to disrupt seizure propagation and has been shown to decrease seizures by up to 73% depending on the target [16,24]. Both VNS and DBS use cycled or continuous nonspecific active stimulation patterns in an open-loop technology that disrupt seizure networks without direct localization of the SOZ [16]. 

While RNS is relatively new, stimulation for seizure abortion as a concept is not. It was first suggested by Penfield and Jasper in 1954 [25]. In a later animal study in 1983 by Psatta et al., epileptic discharges were shown to be disrupted in cats via stimulation immediately after discharge [26]. Stimulations closer in time to the discharge provided a greater benefit, which supported the idea that stimulation in response to seizure activity is superior to continuous stimulation. Lesser et al. applied stimulation to humans in a 1999 study, which showed that cortical stimulation significantly decreased after discharges, and concluded that “electrical stimulation, applied in an appropriate manner at seizure onset, could abort seizures in humans” [27]. Building on these results, Motamedi et al. [28] found that early, targeted stimulation to the SOZ was the most effective at terminating discharges. 

All of these findings culminated in the first responsive stimulator device in humans that showed positive results in preventing seizure activity [29]. However, typical of all early technological devices, this model was very large and cumbersome, consisting of EEG machines and computers kept at the bedside. This provided the impetus to create a device that was small enough to be practical and reliable for patient use. In 2005, Neuropace, Inc. created the RNS device to treat DRE, and subsequent trials have shown its success in adult patients. As the first closed-loop technology offered, RNS both monitors electrical activity and delivers stimulation directly to a target in response to the detected electrographic patterns believed to represent seizure onset [15]. 

## 3. RNS Placement and Proposed Mechanism

### 3.1. Cortical RNS

**Figure 1:**
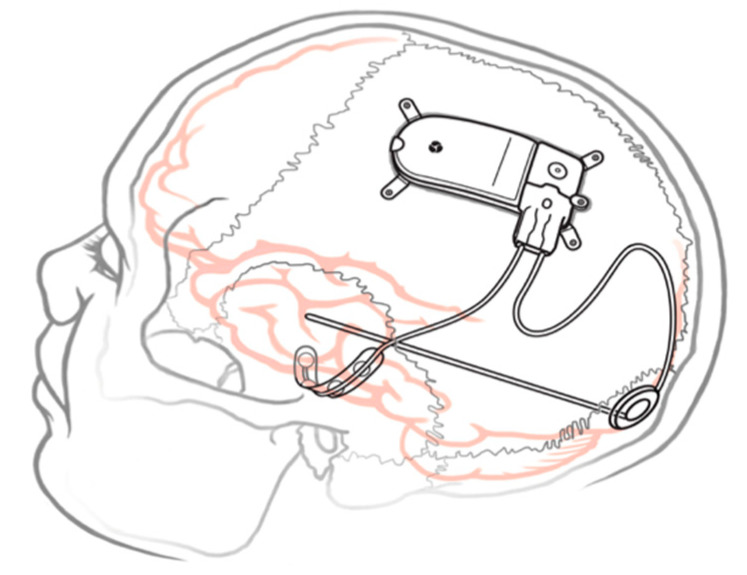
Implanted RNS^®^ System with depth and cortical electrodes. Neurostimulator dimensions: 28 × 60 × 7.7 mm.

Originally, RNS strategies involved implantation of electrodes directly into or onto the epileptogenic zone (EZ) via either depth electrodes or cortical strip electrodes. As a first step, the EZ must be localized, which can be performed using invasive monitoring for MR-negative patients. These electrodes are then connected to a neurostimulator that is implanted into the skull via craniotomy as illustrated in Figure 1 from the RNS user manual [30]. The neurostimulator itself lasts between 6 to 12.4 years (median of 10.8) years before the required replacement; this is dependent on the stimulation and detection utilization [30]. Targeting can be directed to disrupt the onset at the SOZ, propagation of seizure, or both [15,16,31]. Additional leads may be placed at other suspected SOZs that, while not functional, may allow the surgeon to change stimulating lead connections with a simple procedure [32,33]. The option to have depth or surface stimulation contacts increases the versatility of the system and allows for a wide variety of stimulation paradigms. Given the low profile of the system, and the intracranial component in particular, there is still opportunity for more invasive surgical treatments, such as resection or disconnection, should the system prove to be ineffective, epilepsy continues to evolve, or localization or lateralization information is provided by RNS that informs epilepsy surgery [34]. 

The RNS system’s unique design enables detection of, and response to, specific pathologic cortical activity recorded from the local field potential of the electrodes (electrocorticography; ECoG). Data collected by the device can be uploaded wirelessly into a patient data management system for the provider to review on a cloud-based server. Once patterns in ECoG abnormalities have been recognized, the clinician can use the programmer to choose stimulation settings. The RNS device is then programmed to send stimulation pulses in response to those abnormalities with the intent to abort the presumed seizure event. This workflow allows for constant intracranial monitoring, real time analysis, and tailored treatment to a patient’s specific seizure onset patterns, optimizing seizure control [35,36]. 

The RNS system is unique compared to other neuromodulatory procedures in that it delivers stimulation in response to predesignated events and records ECoG surrounding the events. While the exact mechanism of action of the RNS system is not entirely known, the predominant hypothesis is that the responsive stimulation directly inhibits ongoing seizure activity by disrupting generalized seizure propagation [27,37,38]. Kokkinos et al. also identified that in addition to the hypothesized direct inhibition of seizure patterns, there is also potentially direct frequency modulation, resulting in post-stimulus changes to future seizure patterns [37]. Sisterson et al. also suggested that the repeated disruption of network connectivity and synchronization may also allow for decreased seizure severity [39]. This closed-loop technology can both treat individual seizures and affect future seizure patterns, while also collecting data on the patient’s response to provide constant treatment feedback to the care team.

### 3.2. Subcortical RNS

In addition to cortical lead placement, subcortical electrodes can also be used with RNS to reach deeper areas within the seizure network to prevent spreading of electrical activity. This involves drilling a small hole in the skull and stereotactically placing an RNS depth electrode at a predetermined location to a proposed neuromodulation target. The depth electrode is then connected to the recessed neurostimulator placed in the skull, similar to the cortical electrode setup. While the utility and mechanism of the RNS electrode placement outside of the SOZ are not well understood, it is similar in some ways to high-frequency DBS lesioning. Depending on the location of the electrode placement, one can theoretically disrupt seizures at ‘propagation points’ in seizure networks that cannot be reached by cortical electrode placement; however, RNS has the added benefits of closed-loop data collection, responsive stimulation, and potentially lower cognitive side effects from intermittent as opposed to continuous stimulation of DBS [14]. 

Different thalamo-cortical networks are thought to be involved in different epilepsy syndromes [40]. The three typical subcortical nuclei targeted for RNS implantation are the anterior thalamic nucleus (ANT), the centromedian nucleus of the thalamus (CMT), and the pulvinar thalamic nucleus (PNT). The ANT is used to disrupt localized seizures propagating through the hippocampal outflow tract and Papez circuit [41]. The CMT is used as a target for diffuse or multifocal seizure patterns due to its diffuse connectivity to neocortical tissue [42,43]. The PNT can be an attractive target in posterior quadrant epilepsy as this is the largest nucleus in the thalamus, making it an easier target, and it has broad connections to the posterior quadrant [44]. Understanding how each of these networks is involved in seizure generation and propagation is an active area of research that will help dictate RNS targeting in the future [45,46,47].

## 4. Effectiveness and Clinical Indications

Previous trials focused on medical treatment options for epilepsy have found that health-related quality-of-life improvement is seen at a greater than 50% reduction in seizure frequency to assess the utility of a treatment option. While there is some debate as to whether a higher threshold of seizure relief should be used for surgical treatments, 50% continues to dominate as a measure of endpoint success [48].

A pivotal trial with RNS in 2011 showed patients overall experienced a 37% decrease in seizures after a three-month follow-up and an over 53% decrease after two years in a continuation of the original study [35,38]. This led to RNS approval in 2013 by the FDA for the treatment of focal adult DRE [49]. A long-term trial was also performed, which demonstrated continued seizure reduction: 60% after two years, 66% reduction at six years, and a 75% reduction at nine years [31,49,50]. 

In the original study, 50% of these patients had mesial temporal sclerosis with 73% of those being bilateral, indicating that RNS is a good option for these patients. In further studies, the reduction in the targeted seizure type was 70% with a 66% responder rate [31,33]. There were no reported differences in response rates between patients who had unilateral or bilateral mesial temporal SOZs, or those with electrodes in or near the hippocampus. This suggests that the precise location of the electrode on SOZs is not entirely necessary to achieve the therapeutic effect in patients with mesial temporal sclerosis, and that RNS is useful in patients with bilateral epileptogenic foci [31,51]. 

Clinical trials have also investigated the use of RNS in eloquent areas. It demonstrates good seizure control without functional deficits in primary language and motor areas. The reduction in seizure frequency reached 77% in patients with a lesional neocortical SOZ. Overall, there was a 58% reduction in seizures in 55% of patients with neocortical electrode placements in the frontal, parietal, or temporal lobes [52].

Since its Federal Drug Administration (FDA) approval in 2013, RNS has shown that it can be a powerful tool in adult patients with DRE, particularly those in whom surgical therapy would otherwise target an eloquent area, or when there are bilateral targets that would otherwise be inappropriate for resective or disconnective surgery. 

## 5. RNS in Pediatric Patients

While not yet FDA-approved in children, the versatility and success of RNS use in adults has led to off label use in the pediatric population. Currently, the pediatric literature consists of reports and small sample studies; however, these numbers are growing as RNS becomes a more utilized treatment option (Table 1) [32,53,54,55,56,57,58,59,60,61,62]. A recent study from Singh et al. represents the largest pediatric RNS cohort to date with 56 patients across 12 centers [53]. A total of 54% of these patients had a lesion on MRI concerning for a seizure focus. Patients in the study had a variety of previous treatments, including previous surgical intervention (34%) such as resection, disconnection, laser interstitial thermal therapy (LITT), and callosotomy. A total of 67% of their sample had a greater than 50% reduction in seizures, and 10% were seizure-free at 1 year [53]. Only three patients experienced complications. No device failures or postoperative infections were found in this study. There was no difference in response rates when considering the numbers of AEDs, surface or depth electrode combinations, temporal or extratemporal epilepsy, MR-positive or MR-negative epilepsy, or whether patients were evaluated with intracranial monitoring prior to surgery. While the authors did acknowledge limitations in the lack of a control group, examination of confounders, and short follow-up time, this study illustrated a diverse set of conditions in which RNS may produce positive seizure response rates. Typical RNS electrode placement occurs at seizure foci, so considerations must be made as to which cortical or subcortical locations are more amendable to stimulation. In a recent meta-analysis, response rates were found to be 93% in parietal lobe, 88% in frontal lobe, and 50% in temporal lobe epilepsy at 22 months [32]. While sample size was also small (49 patients), the results are supported by similar findings in the nine-year follow-up study in adults [50].

## 6. Complication Rates and Pediatric Considerations

The primary complication reported after RNS implantation in pediatrics is infection [61]. Panov et al. reported infections in three patients, which resulted in depth electrode removal in one patient and entire RNS removal in another [54]. The larger metanalysis from Kerezoudis et al. reported an 8% infection rate [32], while another series reported a 6% infection rate [55]. These data are difficult to interpret, as no large studies exist. There were no reported hemorrhages nor persistent neurologic deficits reported in the pediatric literature. When compared to the pivotal trial in adults, the major adult complications were intracranial hemorrhage at 4.7% and infections at 5.7% [38]. Transient weakness or sensory deficits were minor side effects experienced by patients in all reports. These tended to resolve soon after surgery or with stimulation adjustments. 

A significant consideration unique to pediatrics must involve skull shape. As the RNS device is anchored in the skull, it may change position as the skull grows. Some reports consider both skull diameter and thickness as factors, which may limit the age at which the device may be implanted [54]. However, there have been no reports to suggest RNS placement or efficacy were hindered by skull shape or growth. Studies speculate the youngest suitable patients for RNS would 8 years old, but there are reports of implantation in patients as young as 3 years old [55,56,57]. Similarly, it is expected that the thinner skin of children may put them at higher risk for wound breakdown with the large neurostimulator in place. Long-term follow-up studies will be crucial in determining potential limitations when balancing potential skull development effects with therapeutic success. 

## 7. Future Uses for RNS at Large

The ability to both record information and simultaneously treat seizures with the same device makes the RNS system a powerful tool in drug-resistant seizure disorders. This technology allows for some versatility in how it may be used to better manage these patients as well. As the understanding of these patients evolves over time, so do the potential uses of the RNS. 

Recently, more focus is being placed on using RNS to treat pediatric generalized seizure disorders without a distinct focus. Thalamic nuclei electrode placement has been shown to record ictal activity and prevent seizure propagation. Several reports describe corticothalamic stimulation patterns that involve one lead targeted to a predefined area in the cortex and one to thalamic nuclei [44,63]. Kwon et al. demonstrated sole thalamic stimulation success in two patients [58]. The first patient, a 16-year-old with a history of infantile spasms, was treated with bilateral CMT and frontal leads. With one CMT and one frontal lead connected, the patient saw a 50% decrease in seizure frequency. After 18 months, the frontal lead was deactivated, and the seizure frequencies remained constant. They then connected the second CMT lead during a battery replacement operation. At 26 months follow-up, the patient had a 100% improvement in generalized tonic–clonic seizures, and a 90% improvement in drop attacks was achieved. The second patient was a 12-year-old with Lennox-Gastaut Syndrome who underwent bilateral CMT electrode and fronto-orbital strip electrodes with his RNS system. The CMT electrodes were used without any cortical stimulation, and there was a 95% improvement in seizure frequencies. In another study, authors from a single-center review found seizures to be successfully detected and treated from the thalamus after comparing seizure response rates to ANT, CMT, and PNT [43]. This further corroborates supported results from case reports that thalamic nuclei can be sole targets for generalized seizure syndrome treatment [59,64]. 

Surgery remains the most definitive way to ameliorate seizures in the appropriate patients [65,66,67]. However, RNS may also be used in these patients if surgery has failed or in conjunction with resection of epileptic foci. In a series including three patients with prior surgeries, seizure frequencies were decreased by 50–75% [56]. A larger retrospective review of 27 patients by Panov et al. included 10 with prior surgical interventions, such as anterior temporal lobectomy, frontal disconnection, and corpus callosotomy. Four of these patients achieved a 75–99% seizure reduction [54]. While studies involving RNS implantation after resection are more limited in pediatrics relative to adults, the success rates do not seem to be hindered by prior surgery in either population. 

RNS may also be beneficial as an adjunct therapy with surgery. Singhal et al. demonstrated this in a patient found to have seizures originating from the superior temporal gyrus from focal cortical dysplasia [57]. The authors opted to implant RNS in the same operation because of the difficulty in total resection and the high likelihood of recurrence. At 6-month follow-up, the patient no longer had impaired awareness seizures. Combination treatment may be an important approach in lieu of a second surgery in patients in which complete resection is difficult.

A distinguishing feature of the RNS system is the ability to store ECoG data continuously and provide ambulatory monitoring to better localize SOZs for a later resection. In a study by Hirsch et al., patients with bilaterally implanted leads for mesial temporal lobe epilepsy were analyzed to determine suitability for resection. It was found that 9 patients had unilateral seizures and another 15 had unilateral dominant seizures. These patients all underwent mesial temporal lobe resection, and almost all became seizure-free [34]. Similarly, DiLorenzo et al. reported four patients who were not thought to be candidates for resective surgery due to eloquent cortex involvement. Analysis of continuous RNS data helped to refine the seizure focus to the point where the patient could undergo surgery and became seizure-free [68]. With the many devices implanted to date, large amounts of data can be stored for analysis to study interictal timing, seizure onset locations, and possibly biomarkers for predicting when or where a seizure will occur. Machine learning algorithms are being created to further analyze EEG data to better identify seizure types, timing, and potential markers to predict onset [69]. These examples highlight the potential of RNS as a diagnostic tool in addition to a palliative treatment device. 

## 8. Long-Term Quality of Life with RNS and Effects on the Developing Brain

While anti-seizure medications are the primary treatment option for epilepsy, their long-term adverse effects present a risk–reward trade off that must be taken into consideration [10]. This is particularly true in DRE patients, who may be taking multiple medications. Polytherapy significantly increases the chances of adverse drug reactions [9]. RNS can potentially lessen this concern by obviating the need for multiple or high-dose medications. Reducing the risk of side effects, which in turn may increase general quality of life, will have an enormous impact on patient satisfaction. In adult studies, the Quality of Life in Epilepsy 89 (QOLIE-89) score was significantly higher in surgical patients across all categories, including cognitive function, mental health, and physical health, in addition to treating seizures, which can at least be partially attributed to lower medication side effects [65]. Quality-of-life interviews with pediatric patients and their families also suggest that surgical treatment of seizures has a positive impact on quality of life [70]. More studies need to be performed, however, on measuring the quality of life in pediatric patients that undergo neuromodulation treatment for epilepsy. 

Additionally, multiple studies have demonstrated that, at successive follow-up periods, seizure frequencies have improved in RNS patients [49,50,53]. With the apparent increase in efficacy over time, the case in favor of RNS implantation in pediatric patients continues to strengthen. The exact reason for the increase in efficacy is unknown. However, with the data that are being gathered from the RNS systems, there is growing evidence that the device may play a role in neuroplasticity in these patients. Khambhati et al. showed that stimulation-dependent reorganization of neural networks may be involved via the reorganization of interictal function connectivity [71]. The amount of reorganization correlates with seizure reductions, particularly in the first year of treatment. This timeline agrees with reports that show increases in seizure reduction after the first year of treatment [15,35,50,60]. Pediatric patients may particularly benefit from this as the developing brain displays a higher degree of plasticity [72].

As RNS has not been approved for pediatric use, there is only enough data and experience in the literature to speculate on future RNS uses. While clinical trials may be difficult, these will become essential to better understand and broaden the application of RNS. There are currently three ongoing trials. The RESPONSE study aims to determine the safety and efficacy of RNS as an adjunctive therapy for individuals 12–17 years old with focal DRE. The NAUTILUS trial will determine if RNS is effective in patients that are 12 years and older with drug-resistant idiopathic generalized epilepsy. Finally, the LGS study will determine if RNS can help reduce generalized seizures in patients 12 and older with Lennox-Gastaut Syndrome.

## 9. Conclusions

DRE is a common disorder in children, for which surgical options have become more available over time. Surgical resection and disconnection provide the most direct and potentially more reliable benefit with seizure freedom. However, neuromodulation, and more specifically RNS, has provided a potential minimally invasive treatment option for patients with lesions in eloquent areas, are poor surgical candidates, or have bilateral seizure foci. The ability of RNS to gather information via intracranial EEG recordings while also treating with stimulation allows for not only individualized treatment but also a better understanding of the peri-ictal period and epilepsy syndromes in general. RNS may also have a positive effect on neuroplasticity, allowing for seizure rates to continue to improve over time for younger patients. Several clinical trials are ongoing for pediatric RNS usage in children 12 years and older. 

## Figures and Tables

**Table 1:** Summary of the Literature Reporting the use of RNS in Pediatric Patients.

Authors	Study Type	Title	Summary Points
Singh (2023) [53]	Case series	Responsive neurostimulation in drug-resistant pediatric epilepsy findings from the Epilepsy Surgery Subgroup of the Pediatric Epilepsy Research Consortium	Largest multicenter pediatric sample (*n* = 56). 67% with >50% reduction in seizures; 10% seizure-free at 1 year.
Kerezoudis (2022) [32]	Meta-analysis	Safety and efficacy of responsive neurostimulation in the pediatric population: evidence from institutional review and patient level meta-analysis	8 studies (*n* = 49) reviewed with 80% responders and 75% median seizure reduction. Most common locations for implantation were frontal and mesial temporal lobe; 8% infection rate.
Curtis (2022) [61]	Case series	Responsive neurostimulation for pediatric patients with drug-resistant epilepsy: a case series and review of the literature	*n* = 20. Cohort with varied semiology and both eloquent and thalamic electrode implantation. Similar complication profile to the adult literature.
Hartnett (2022) [62]	Case series	Responsive neurostimulation device therapy in pediatric patients with complex medically refractory epilepsy	*n* = 8, 50% with previous surgery for epilepsy. All achieved >50% seizure reduction.
Beaudreault (2022) [43]	Case series	Responsive neurostimulation targeting the anterior, centromedian, and pulvinar thalamic nuclei and the detection of electrographic seizures in pediatric and young adult patients	*n* = 17 (mean 16.5 years old) underwent thalamic depth electrode placement with or without cortical strip leads. Thalamic leads alone were able to detect and prevent propagation similarly to combined thalamic and cortical strip setup.
Nagahama (2021) [55]	Case series	Real-world preliminary experience with responsive neurostimulation in pediatric epilepsy: a multicenter retrospective observational study	*n* = 35 identified from 5 centers (age 3–25 years old). 50% had >50% reduction in seizures. No complications in pediatric patients, 3 complications in young adults. RNS can be used in patients as young as 3 years old.
Welch (2021) [59]	Case report	Responsive neurostimulation of the centromedian thalamic nucleus for the detection and treatment of seizures in pediatric primary generalized epilepsy	16 year old male with primary generalized epilepsy with 75% seizure reduction following bilateral CMT RNS placement and complete resolution of absence seizures at 6 months.
Panov (2020) [54]	Retrospective review	Safety of responsive neurostimulation in pediatric patients with medically refractory epilepsy	Among 27 consecutive pediatric RNS placements. Three patients had infections, but no other complications at 22 months. Seizure frequency improved for all patients.
Kwon (2020) [58]	Case series	Centromedian thalamic responsive neurostimulation for Lennox-Gastaut epilepsy and autism	Two cases of Lennox-Gastaut patients who experienced 75–99% seizure reduction at 1 year after CMT RNS placement.
Bercu (2020) [56]	Case series	Responsive neurostimulation for refractory epilepsy in the pediatric population: a single-center experience	Six patients with focal epilepsy who underwent RNS experienced improvement in seizure frequency and changes in semiology as well.
Kokoszka (2018) [60]	Case report	Treatment of medically refractory seizures with responsive neurostimulation: 2 pediatric cases	14 year old with bilateral cortical dysplasia with 80–90% reduction in seizure frequency with cortical strip RNS. Corticothalamic treatment reduced seizures another 50%; 9 year old with cortical leads placed at focus resulted in behavioral improvement and >80% seizure reduction.
Singhal (2018) [57]	Case report	Single report of successful RNS placement in pediatric patient	16 year old male with simultaneous subtotal resection of cortical dysplasia and RNS placement; 100% reduction in impairing seizures at 6 months

## Data Availability

Not applicable.
